# Ex Vivo Expansion of Human Limbal Epithelial Cells Using Human Placenta-Derived and Umbilical Cord-Derived Mesenchymal Stem Cells

**DOI:** 10.1155/2017/4206187

**Published:** 2017-08-15

**Authors:** Sang Min Nam, Yong-Sun Maeng, Eung Kweon Kim, Kyoung Yul Seo, Helen Lew

**Affiliations:** ^1^Department of Ophthalmology, CHA Bundang Medical Center, CHA University, Seongnam, Republic of Korea; ^2^Department of Obstetrics and Gynecology, Institute of Women's Life Medical Science, Yonsei University College of Medicine, Seoul, Republic of Korea; ^3^Department of Ophthalmology, Institute of Vision Research, Severance Hospital, Yonsei University College of Medicine, Seoul, Republic of Korea; ^4^Corneal Dystrophy Research Institute, Yonsei University College of Medicine, Seoul, Republic of Korea

## Abstract

Ex vivo culture of human limbal epithelial cells (LECs) is used to treat limbal stem cell (LSC) deficiency, a vision loss condition, and suitable culture systems using feeder cells or serum without animal elements have been developed. This study evaluated the use of human umbilical cord or placenta mesenchymal stem cells (C-MSCs or P-MSCs, resp.) as feeder cells in an animal/serum-free coculture system with human LECs. C-/P-MSCs stimulated LEC colony formation of the stem cell markers (p63, ABCG2) and secreted known LEC clonal growth factors (keratinocyte growth factor, *β*-nerve growth factor). Transforming growth factor-*β*-induced protein (TGFBIp), an extracellular matrix (ECM) protein, was produced by C-/P-MSCs and resulted in an increase in p63^+^ ABCG2^+^ LEC colonies. TGFBIp-activated integrin signaling molecules (FAK, Src, and ERK) were expressed in LECs, and TGFBIp-induced LEC proliferation was effectively blocked by a FAK inhibitor. In conclusion, C-/P-MSCs enhanced LEC culture by increasing growth of the LSC population by secreting growth factors and the ECM protein TGFBIp, which is suggested to be a novel factor for promoting the growth of LECs in culture. C-/P-MSCs may be useful for the generation of animal-free culture systems for the treatment of LSC deficiency.

## 1. Introduction

The limbus, where corneal epithelial stem cells reside, is the narrow zone between the cornea and the bulbar conjunctiva. Damage to the limbus results in limbal stem cell deficiency (LSCD), which causes severe vision loss by painful opacification of the otherwise transparent cornea. For LSCD treatment, transplantation of in vitro cultured limbal epithelial cell (LEC) sheets grown on various culture substrates has shown success in terms of ocular surface reconstruction and improved vision [1]. Therefore, it will be essential to determine a suitable culture system using different carriers of the sheet, culture medium, or feeder layer.

Murine 3T3 feeder layer cells increase the colony-forming efficiency of LECs and produce a robust sheet by unclear mechanisms [2]. However, the use of xenologic 3T3 cells may expose human LECs to mouse pathogens, presenting ethical and safety issues. To avoid xenogenic contamination, human dermal fibroblasts, human bone marrow mesenchymal stem cells (BM-MSCs), human limbal mesenchymal cells, and human adipose tissue-derived MSCs have been used as replaceable feeder cells [2–5]. Human MSCs express various genes that maintain or promote the proliferation of LECs, for example, pleiotrophin, epiregulin, cystatin C, hepatocyte growth factor (HGF), keratinocyte growth factor (KGF), and insulin-like growth factor 1a in adipose tissue-derived MSCs and KGF, HGF, and N-cadherin in BM-MSCs [2, 3]. Human umbilical cord or placenta MSCs (C-MSCs or P-MSCs, resp.) may also be candidate feeder cells but, to the best of our knowledge, have not been tested in LEC culture.

Compared with BM-MSCs, a collection of human C-/P-MSCs is noninvasive and considered ethically acceptable, because the human placenta is discarded postpartum [6]. MSCs are abundant in the placenta but rare in the adult bone marrow [6]. In addition, the number of BM-MSCs significantly decreases with age [6]. C-/P-MSCs can be obtained efficiently, because P-MSCs grow faster and more robustly in culture than do BM-MSCs, and a substantial number of C-MSCs can be obtained after several passages [7, 8]. A feeder layer of C-MSCs also showed a nontumorigenic effect in embryonic stem cells by downregulating c-myc signaling [9].

With LSCD LEC sheet therapy, the percentage of p63^+^ cells in culture is positively associated with the clinical outcome [10]. p63 sustains the proliferative potential of limbal stem cells (LSCs) and is expressed in holoclones [10–12]. ATP-binding cassette subfamily G member 2 (ABCG2) is the most useful marker of LSCs [12]. In this study, the potential of C-/P-MSCs in LEC culture was assessed according to p63^+^ and ABCG2^+^ colony formation. In addition, a new effective factor, transforming growth factor-*β*-induced protein (TGFBIp), was shown to be secreted by C-/P-MSCs, and a potential signaling pathway stimulating LEC proliferation is suggested.

## 2. Materials and Methods

### 2.1. LEC Culture

Limbal epithelial sheets were isolated from human corneoscleral rims after penetrating keratoplasty. This study was approved by the Institutional Review Board of Severance Hospital (Seoul, Republic of Korea). A human corneoscleral rim was incubated at 4°C for 10 h in CnT-PR medium (CELLnTec, Bern, Switzerland) containing 50 mg/mL dispase II (Roche, Indianapolis, IN, USA) and 100 mM sorbitol (Sigma-Aldrich, St. Louis, MO, USA). Under a dissection microscope, a loose limbal epithelial sheet was separated using a spatula, as described previously [13]. The limbal epithelial sheet was cultured on a 1 : 20 diluted Matrigel (Sigma-Aldrich)-coated plate in CnT-PR medium.

### 2.2. MSC Culture

P-MSCs (passage 6) and C-MSCs (passage 5) were provided by CHA Biotech (Seongnam, Republic of Korea). Preparation and characterization of both cell types have been described previously [14]. MSCs were cultured on a 150 mm culture dish in minimum essential medium- (MEM-) alpha (Gibco, Carlsbad, CA, USA) and 10% fetal bovine serum (FBS; Gibco).

### 2.3. Preparation of MSC-Conditioned Medium and the Colony-Forming Unit Assay

Confluent P-MSC or C-MSC cultures were grown in MEM-alpha without FBS for 24 hours, and the media were collected and centrifuged to remove cells and debris. The conditioned medium was added to CnT-PR medium at a 1 : 1 ratio. LECs (4 × 10^3^) were seeded on a 1 : 20 diluted Matrigel-coated 60 mm dish containing conditioned medium and incubated for 10 days. The colonies were then fixed with 4% paraformaldehyde and stained with hematoxylin and eosin. The number of colony-forming units (CFU) in each dish was counted in four different microscopic fields of view. Experiments were performed in triplicate for each medium condition.

### 2.4. Coculture of MSCs and LECs

Cell culture inserts (Transwell; polycarbonate, 0.4 *μ*m pore size; Corning, NY, USA) were coated with 1 : 20 diluted Matrigel, and 3 × 10^3^ LECs were seeded on the inserts. In addition, 7 × 10^4^ P-MSCs or C-MSCs were seeded as feeder cells in the bottom well of the paired wells and cocultured with LECs in CnT-PR medium for 10 days. Both P-MSCs and C-MSCs were tested in experiments using coculture. A CFU assay for LECs was performed as described above.

### 2.5. Immunocytochemical Analysis of LEC Colonies

LEC colonies were washed in phosphate-buffered saline (PBS), blocked with 5% donkey serum in an antibody dilution buffer consisting of PBS and 0.1% Triton X-100, incubated overnight with the primary antibody (p63 [Cell Signaling, Beverly, MA, USA] or ABCG2 [Abcam, Cambridge, MA, USA]) at 4°C, and labeled with a fluorescein-conjugated secondary antibody (Molecular Probes, Leiden, Netherlands). The colonies were then observed under a fluorescence microscope (Olympus, Tokyo, Japan).

### 2.6. Proteins Secreted by the MSCs

Protein levels in the MSC and LEC coculture medium were measured using enzyme-linked immunosorbent assay (ELISA) kits: human KGF quantikine ELISA kit (DKG00, R&D Systems, Minneapolis, MN, USA), human *β* IG H3 ELISA kit (TGFBI) (ab155426; Abcam), human EGF ELISA kit (ELH-EGF-001, THP; RayBiotech, Vienna, Austria), and human NGF-*β* ELISA kit (K0331220; Koma Biotech, Seoul, Republic of Korea). ELISAs were performed in triplicate for each sample, and coculture was performed in triplicate for each type of MSC.

### 2.7. Effect of TGFBIp on LEC Growth and Colony Formation

The 3-(4,5-dimethylthiazol-2-yl)-2,5-diphenyltetrazolium bromide (MTT) assay was used to evaluate LEC proliferation following TGFBIp treatment. LECs were seeded at 2 × 10^4^/well in 96-well culture plates in CnT-PR medium. After 24 hours, the cells were then treated with human TGFBIp (Sino Biological Inc., 10569-H08H, Beijing, China) at different concentrations ranging from 0–10 *μ*g/mL. Following TGFBIp treatment (24, 48, and 72 h), 20 *μ*L MTT labeling reagent (5 mg/mL) was added to each well. The media were removed 4 hours later, 150 *μ*L dimethyl sulfoxide (DMSO) was added to each well, and the absorbance was measured at 570 nm.

A CFU assay was performed for LECs treated with TGFBIp. LECs (4 × 10^3^) were seeded on a 1 : 20 diluted Matrigel-coated 60 mm dish containing CnT-PR medium and TGFBIp (10 *μ*g/mL). After 10 days of culture, the colony numbers were counted and the expression of p63 and ABCG2 was analyzed as described above.

### 2.8. Western Blotting of Signaling Pathway Members Induced by TGFBIp

LECs (2 × 10^5^/well) were seeded onto 60 mm plates. After 24 hours, cells were treated with TGFBIp (10 *μ*g/mL) for various periods (5, 10, 15, 30, and 60 minutes). Cells were harvested using radio immunoprecipitation assay buffer; the resulting cell lysates were subjected to sodium dodecyl sulfate-polyacrylamide gel electrophoresis, and proteins from the gel were transferred onto polyvinylidene difluoride membranes. The blocked membranes were incubated with the appropriate primary antibody (anti-human, phosphorylated Src, phosphorylated AKT, extracellular signal-regulated kinase [ERK], phosphorylated ERK [pERK], focal adhesion kinase [FAK], or phosphorylated FAK [1 : 1000 dilution, Cell Signaling Technology Inc., Danvers, MA, USA]), and the immunoreactive bands were visualized using an enhanced chemiluminescence immunoblotting system (GE Healthcare, Buckinghamshire, UK). The intensity of a protein band was quantified by densitometry of immunoblots.

### 2.9. FAK Inhibitor and LEC Proliferation Assay

LECs were seeded at 2 × 10^4^/well in 96-well culture plates. The cells were incubated in CnT-PR medium for 24 hours and preincubated for 40 minutes with or without 1 *μ*M CAS 4506-66-5 (FAK inhibitor 14, Sigma-Aldrich). The cells were then treated with 10 *μ*g/mL human TGFBIp (Sino Biological Inc., 10569-H08H). Following TGFBIp treatment (24, 48, and 72 hours), 20 *μ*L MTT labeling reagent (5 mg/mL) was added to each well. The media were removed 4 hours later, 150 *μ*L DMSO was added to each well, and the absorbance was measured at 570 nm.

## 3. Results

### 3.1. Promotion of p63^+^ ABCG2^+^ LEC Colonies by C- or P-MSCs

**Figure 1:**
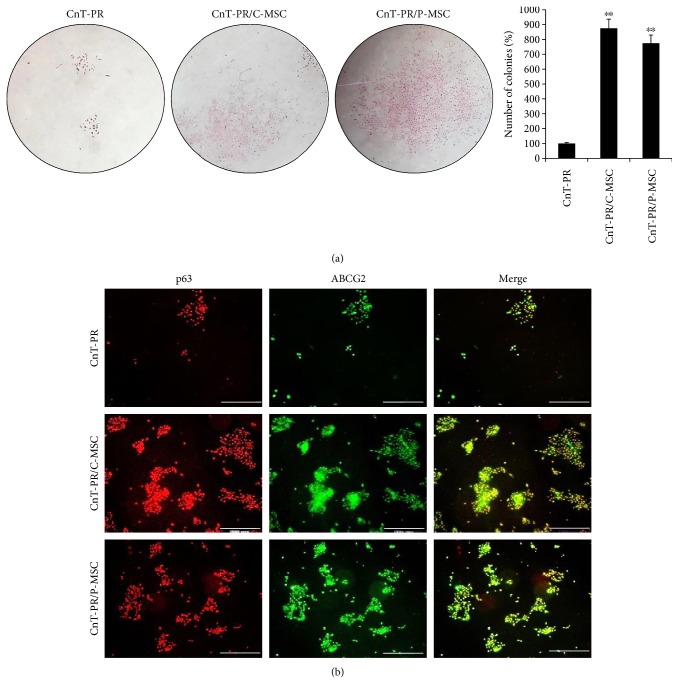
Colony-forming unit assay of conditioned medium from C-MSC or P-MSC. Microscopic analysis following hematoxylin and eosin (H&E) staining (a; magnification: ×10) and immunofluorescence analysis of p63 and ABCG2 (b; white bar indicates 500 *μ*m) are shown. Error bars represent the standard deviation of the mean. ^∗∗^*P* < 0.01, compared with the control medium, CnT-PR (Student's *t*-test). C-MSC: umbilical cord-derived mesenchymal stem cell; P-MSC: placenta-derived mesenchymal stem cell; ABCG2: ATP-binding cassette subfamily G member 2.

**Figure 2:**
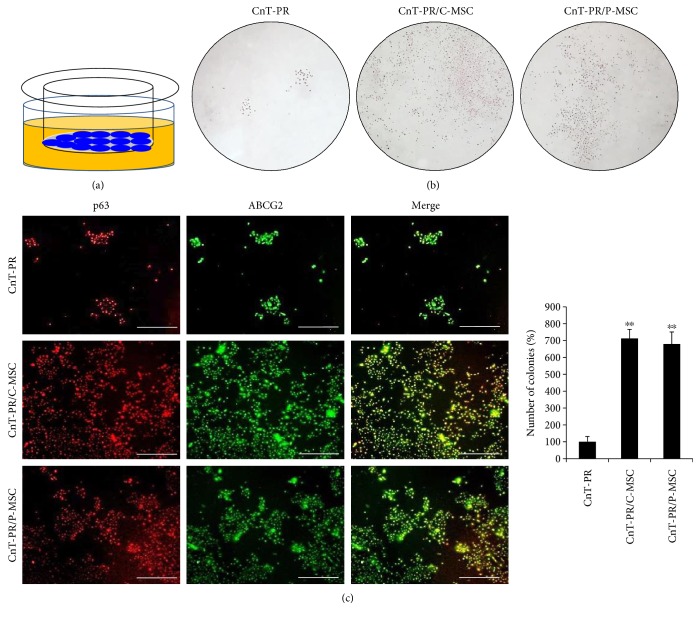
Coculture of MSCs and limbal epithelial cells (LECs) and colony-forming unit assay. Schematic of the coculture system containing LECs (blue in the insert) and MSCs (not shown). Yellow indicates medium. (a) Microscopic analysis following H&E staining (b; magnification: ×10) and immunofluorescence of p63 and ABCG2 (c; white bar indicates 500 *μ*m) are shown. Error bars represent the standard deviation of the mean. ^∗∗^*P* < 0.01, compared with the control medium, CnT-PR (Student's *t*-test). C-MSC: umbilical cord-derived mesenchymal stem cell; P-MSC: placenta-derived mesenchymal stem cell; ABCG2: ATP-binding cassette subfamily G member 2.

C- or P-MSCs were tested in two different systems, conditioned medium and coculture systems. First, conditioned media from both C-MSC and P-MSC cultures increased the number of LEC colonies compared with the basal medium, CnT-PR (Figure 1). In addition, almost all of the colonies were positive for p63 and ABCG2 (Figure 1). Because ABCG2 is a marker of LSCs and p63 is correlated with positive clinical outcomes, some factors in the conditioned medium may promote a positive clinical effect by promoting the growth of p63^+^ and ABCG2^+^ cells. A Transwell coculture system was designed: in this convenient system, C-/P-MSCs did not contact LECs directly, but LECs were continuously exposed to factors secreted from C-/P-MSCs (Figure 2). In this system, elements were directly transferred from C-/P-MSCs to LECs. In both C-MSC and P-MSC coculture systems, the number of colonies increased in comparison to that of colonies in CnT-PR medium, and nearly all of the colonies were positive for p63 and ABCG2 (Figure 2). However, the number of colonies generated was not greater in the coculture system compared with conditioned medium (Figures 1 and 2). Because only CnT-PR medium was used in the coculture system, the increase in the number of LEC colonies appeared to be caused by factors secreted by C-/P-MSCs. In contrast, the conditioned medium consisted of MEM-alpha, CnT-PR, and elements secreted by C-/P-MSCs. Therefore, secreted factors in the conditioned medium and the coculture system promoted the formation of p63^+^ ABCG2^+^ LEC colonies. Although both C-MSCs and P-MSCs were cultured in CnT-PR medium without serum in the coculture system, their functions were comparable when grown in MEM-alpha medium with serum.

### 3.2. *β*-NGF, KGF, and TGFBIp Were Present at Higher Levels in the Coculture System

**Figure 3:**
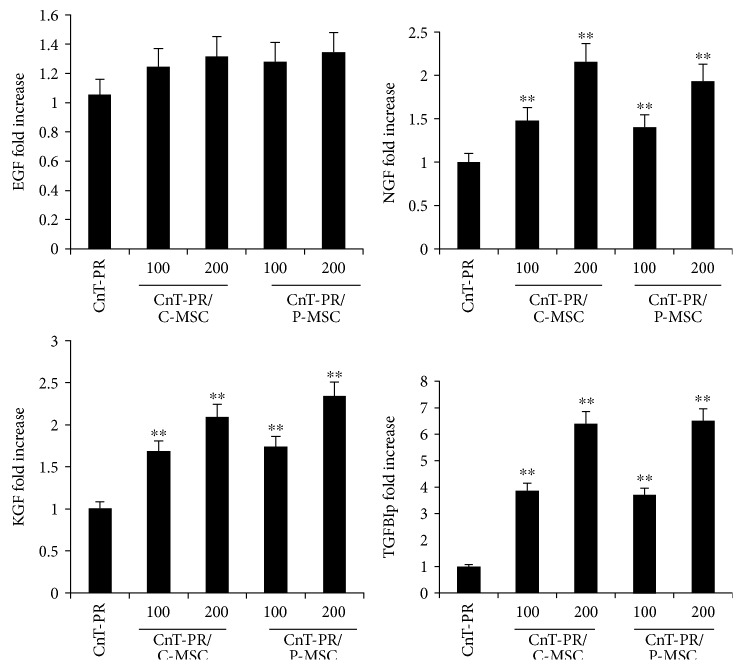
Human protein levels in the supernatant of the coculture medium, detected by the enzyme-linked immunosorbent assay. C-MSCs or P-MSCs were cultured with limbal epithelial cells. The control medium was CnT-PR, and each supernatant was tested in two different volumes: 100 *μ*L and 200 *μ*L. ^∗∗^*P* < 0.01, compared with the control medium, CnT-PR (Student's *t*-test). Error bars represent the standard deviation of the mean. C-MSC: umbilical cord-derived mesenchymal stem cell; P-MSC: placenta-derived mesenchymal stem cell; EGF: epidermal growth factor; NGF: nerve growth factor; KGF: keratinocyte growth factor; TGFBIp: transforming growth factor-*β*-induced protein.

To determine the factors secreted by C-/P-MSCs, the supernatant obtained from the coculture medium was analyzed by ELISA to detect human epidermal growth factor (EGF), KGF, *β*-nerve growth factor (*β*-NGF), and TGFBIp. EGF, KGF, and *β*-NGF stimulate clonal growth of LSCs, and TGFBIp was chosen as a novel candidate [15]. In both C-MSC and P-MSC coculture media, EGF was not significantly elevated compared with that in CnT-PR medium alone (Figure 3). In contrast, KGF, *β*-NGF, and TGFBIp were detected at higher levels in both C-MSC and P-MSC coculture media compared with CnT-PR medium alone (Figure 3). The concentration of TGFBIp was remarkably high compared with that of KGF and *β*-NGF in both C-MSC and P-MSC coculture media (Figure 3). Based on these findings, KGF, *β*-NGF, and TGFBIp, but not EGF, may have been responsible for the increase in p63^+^ ABCG2^+^ LEC colonies.

### 3.3. TGFBIp Promoted Colony Formation and Proliferation of LECs via FAK and ERK Signaling

**Figure 4:**
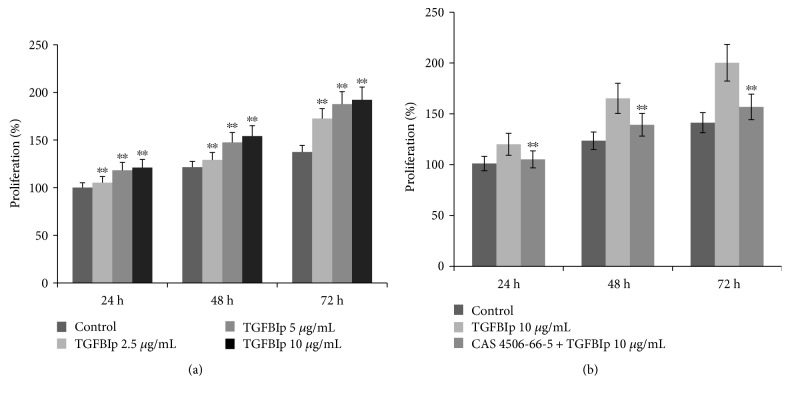
Effect of TGFBIp on limbal epithelial cell (LEC) proliferation. LECs were incubated with TGFBIp (0–10 *μ*g/mL) for 72 h (a). LECs were preincubated with or without the FAK inhibitor CAS 4506-66-5 (1 *μ*M) and then stimulated with TGFBIp (10 *μ*g/mL) for 72 h (b). Cell proliferation was assessed by the MTT assay. Error bars represent the standard deviation of the mean. ^∗∗^*P* < 0.01, compared with the control medium, TGFBIp 0 *μ*g/mL (Student's *t*-test). TGFBIp: transforming growth factor-*β*-induced protein.

**Figure 5:**
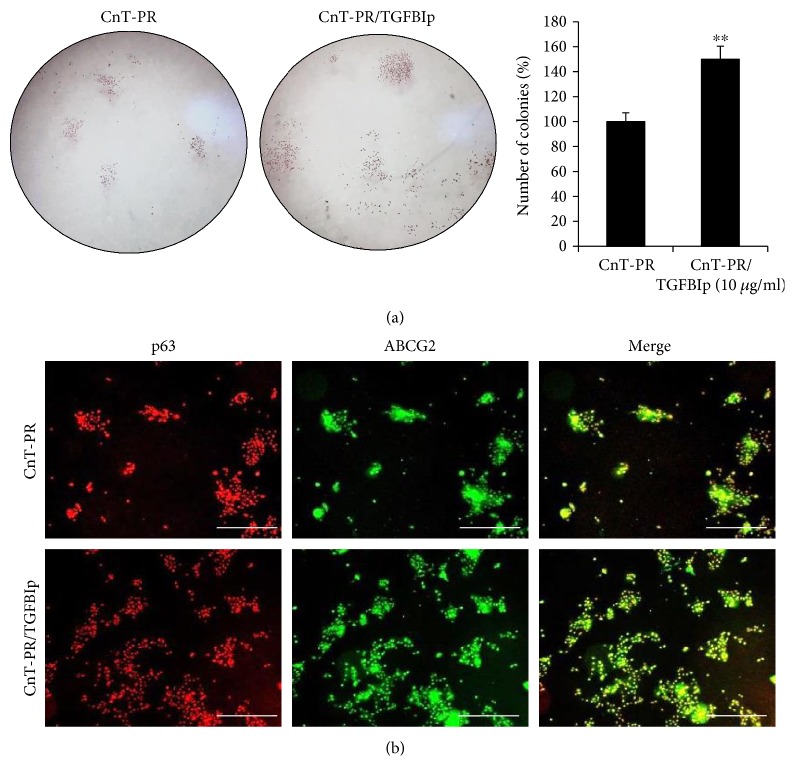
Colony-forming unit assay of limbal epithelial cell (LEC) after treatment with TGFBIp. Microscopic analysis following H&E staining (a; magnification: ×10) and immunofluorescence of p63 and ABCG2 (b; white bar indicates 500 *μ*m) are shown. Error bars represent the standard deviation of the mean. ^∗∗^*P* < 0.01, compared with the control medium, CnT-PR (Student's *t*-test). TGFBIp: transforming growth factor-*β*-induced protein.

To confirm the effect of TGFBIp, a LEC proliferation assay was performed, and the results revealed that TGFBIp promoted proliferation of LECs in a dose-dependent manner up to 10 *μ*g/mL at three different time points (Figure 4(a)). TGFBIp was added to CnT-PR medium at 10 *μ*g/mL, and the TGFBIp-containing medium resulted in more colonies than did the control medium; the majority of which were p63^+^ and ABCG2^+^ (Figures 5(a) and 5(b)).

**Figure 6:**
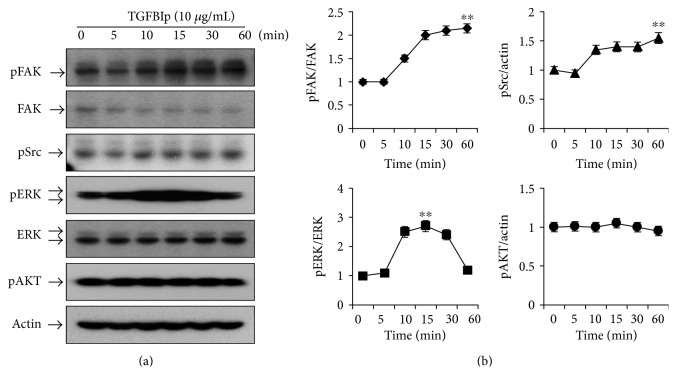
Effects of TGFBIp on the FAK, Src, and ERK signaling pathways in limbal epithelial cells (LECs). Representative Western blotting images (a) and densitometric analyses (b). LECs were treated with TGFBIp (10 *μ*g/mL) for the indicated periods of time, and cell lysates were subjected to Western blot analysis. The relative ratios were normalized by arbitrarily setting the phosphorylation ratio at 0 minute as 1. Analyses were performed in triplicate. Error bars represent the standard error of the mean. ^∗∗^*P* < 0.01, compared with the ratio at 0 minute (Student's *t*-test). TGFBIp: transforming growth factor-*β*-induced protein; FAK: focal adhesion kinase; ERK: extracellular signal-regulated kinase.

To determine which signaling pathways are involved in TGFBIp-induced LEC proliferation, different intracellular signaling molecules were analyzed. TGFBIp interacts with integrins with multiple motifs, including the C-terminal RGD motif, and activates integrin-associated proteins, such as FAK and Src [16]. As a result, FAK promotes cell proliferation via ERK signaling or cell survival via AKT activation [16]. The expression of FAK, SRC, ERK, and AKT were evaluated by Western blotting following incubation with TGFBIp-containing (10 *μ*g/mL) CnT-PR medium. Phosphorylation of FAK, SRC, and ERK was increased by TGFBIp stimulation, while AKT phosphorylation was unaffected (Figure 6). To investigate the significance of the FAK signaling pathway, LECs were pretreated with a FAK inhibitor (CAS 4506-66-5) and stimulated with TGFBIp. LEC proliferation by TGFBIp was effectively blocked by CAS 4506-66-5 at three different time points (Figure 4(b)). Overall, TGFBIp stimulated LEC proliferation, which was suppressed by CAS 4506-66-5, and the formation of p63^+^ ABCG2^+^ LEC colonies, suggesting that TGFBIp may act on LECs via integrins.

## 4. Discussion

Factors secreted by human C-/P-MSCs promoted the formation of p63^+^ ABCG2^+^ colonies in LEC culture. The levels of KGF, *β*-NGF, and TGFBIp were elevated in C-/P-MSC coculture media, and TGFBIp stimulated LEC proliferation via the FAK, Src, and ERK signaling pathways.

CnT-PR is a serum-free medium developed for progenitor cell-targeted growth with good colony-forming ability [15]. However, in LEC culture, serum-free CnT-PR is unsuccessful for LSC transplantation, as serum support seems to be essential [17, 18]. Vitronectin and fibronectin, ECM proteins present in FBS, are important for the initial attachment of LECs [19]. For clinical use, animal-free culture techniques have been developed using autologous serum, and these have shown promising clinical results [20]. In this study, serum was removed from the C-/P-MSC culture medium 24 hours before adding it to CnT-PR medium to generate the conditioned medium. Coculture with C-/P-MSCs was also performed under serum-free conditions as only CnT-PR medium was used. Under both serum-free conditions, the number of the p63^+^ ABCG2^+^ LEC colonies was increased compared with that under the use of CnT-PR medium alone, which implied that C-/P-MSCs enhanced the LEC culture system for LSCD treatment in the absence of serum (Figures 1 and 2).

Human epidermal keratinocytes generate three types of clonogenic cells, holoclones, meroclones, and paraclones, which have different proliferative capacities [21]. Holoclone-forming cells have the ability to self-renew and high proliferative potential; they are human squamous epithelial stem cells located in the limbus but not in the central cornea [10, 21]. p63 is a marker of holoclones, and quantitative immunodetection of p63 is used as a validation method before grafting in LSCD patients [10, 22]. Success of LEC transplantation is associated with the percentage of p63-positive cells (p63%) in culture; the success rate is 78% if p63% > 3% but only 11% if p63% ≦ 3% [10]. Therefore, the increase in p63^+^ LEC colony number by C-/P-MSCs could improve LEC transplantation.

3T3 fibroblasts have been introduced as feeder cells [23]. Because 3T3 fibroblasts are effective without directly contacting LECs, soluble factors derived from 3T3 fibroblasts may be involved in the promotion of LSCs [24]. Hence, the Transwell coculture system may be clinically applicable and potentially involve soluble factors from the MSCs (Figure 2). In addition, the Transwell coculture system helps to avoid mixing problems with feeder cells because transplantable LECs on the insert can be cultured separately. The importance of soluble factors was reaffirmed by the C-/P-MSC-conditioned medium (Figure 1). In comparison with conditioned medium, the coculture system provides fresh MSC-derived factors to the LECs. However, the coculture conditions may not be perfect for MSCs, for which serum-free CnT-PR medium was used. For the conditioned medium, C-/P-MSCs were cultured with serum and MEM-alpha. As a result, the colony formation ability of the coculture system was not superior but comparable with that of the conditioned medium (Figures 1 and 2).

The soluble factors KGF and *β*-NGF, but not EGF, were present at higher levels in the coculture medium compared with the CnT-PR control medium (Figure 3()). KGF and EGF stimulate clonal growth of human LECs more than do HGF and other growth factors [15]. Expression of KGF in human MSCs has been reported in LEC culture, but EGF and *β*-NGF have not been investigated [2, 3]. KGF is highly expressed in limbal fibroblasts, and the basal layers of the limbal epithelium express the KGF receptor [25]. KGF increases p63 expression in human LECs, and human LSCs may require KGF expression to maintain an undifferentiated state [25]. However, KGF does not inhibit the differentiation of LECs, which may be beneficial for the culture of LEC sheets that include both undifferentiated and differentiated cell layers [25, 26]. In contrast, EGF inhibits the expression of differentiation markers in corneal epithelial cells [26]. The NGF receptor, TrkA, is also expressed in limbal epithelial basal cells, and *β*-NGF exhibits an additive effect with EGF on the promotion of LEC growth rate [15]. *β*-NGF signaling favors LSC survival and is important for the expansion of limbal epithelial progenitor cells; a high level of NGF is present in the human amniotic membrane, and LEC expansion on the amniotic membrane is significantly retarded by blocking NGF signaling [27].

TGFBIp was detected at much higher concentrations in C-/P-MSC coculture medium compared with CnT-PR control medium, and it promoted LEC colony formation and proliferation (Figures 3, 4(a), and 5). To the best of our knowledge, the effect of TGFBIp on LEC colony formation has not yet been reported. TGFBIp is an ECM protein detected in various cell types, including MSCs and corneal epithelial cells [28–30]. TGFBIp participates in diverse processes including cell adhesion and migration and contributes to wound healing in human corneal epithelial cells [28, 30]. TGFBIp has multiple integrin-binding motifs including Arg-Gly-Asp (RGD), NKDIL (sequence of peptide), and EPDIM (sequence of peptide) and interacts with other ECM molecules, such as collagen, fibronectin, laminin, or glycosaminoglycan [29, 30]. Therefore, TGFBIp would be able to function as a linker protein connecting the ECM and integrins. LECs express several integrins that can interact with TGFBIp, such as *α*3*β*1, *α*v*β*5, and *α*6*β*4 [30, 31]. TGFBIp may create favorable conditions for LEC attachment during culture, resulting in increased colony formation.

The LEC integrins *α*3*β*1 and *α*6*β*4 may function in cell survival and proliferation when activated by TGFBIp [31]. Integrins cooperate with other growth factors or cytokine receptors by transactivation, coordination, modulation, and compartmentalization [32]. In addition, *β*1 and *β*4 are highly expressed in LSCs and may be important for maintaining the stem cell population [31, 33]. Integrins play an important role in the regulation of stem cell function including stem cell proliferation, self-renewal, and cell division orientation [33]. Consequently, TGFBIp may promote LEC cell proliferation and p63^+^ ABCG2^+^ colony formation.

In intracellular integrin signaling, FAK associates with integrin and plays a key role in the formation of a signaling complex with other molecules [34]. The activated dual kinase FAK-Src complex functions to promote cell migration, proliferation, and survival [16]. Crk-associated substrate and c-Jun N-terminal kinase (JNK) are involved in cell migration, ERK and cyclin D1 are important for cell cycle progression, and phosphatidylinositol 3-kinase (PI3K) and AKT are involved in FAK-dependent cell survival [16, 34]. In the TGFBIp-dependent pathway, *α*v*β*5 integrin triggers phosphorylation and activation of FAK, paxillin, AKT, and ERK and mediates the adhesion and migration of vascular smooth muscle cells [35]. Coculture with mouse embryonic stem cells enhances the adhesion, migration, and proliferation of rabbit corneal epithelial cells via the integrin *β*1-FAK-AKT pathway [36]. In the present study, TGFBIp activated FAK, Src, and ERK signaling, but not AKT signaling, in human LECs (Figure 6). Because TGFBIp-dependent LEC proliferation was effectively suppressed by a FAK inhibitor (Figure 4(b)), FAK activation by integrin might be an essential step for TGFBIp action in LECs, although the importance of Src and ERK remains to be assessed. This study showed that TGFBIp was secreted by C-/P-MSCs and TGFBIp activity in LECs required FAK activation. ERK has been shown to regulate the migration of immortalized human corneal epithelial cells during wound healing by modulating the phosphorylation of FAK and paxillin [37]. Therefore, ERK may not be a downstream mediator of FAK.

## 5. Conclusions

Human C-/P-MSCs supported the LSC population in LEC culture by secreting growth factors and ECM proteins such as TGFBIp. We have shown that TGFBIp regulates LECs possibly via integrin signaling. Human C-/P-MSCs can be obtained safely and efficiently and may help in the development of an animal-free LEC culture system for the treatment of LSCD.
